# Perspectives on potential pharmacist prescribing in an outpatient dialysis center: qualitative interviews with patients and clinicians

**DOI:** 10.1007/s11096-025-02084-x

**Published:** 2026-01-17

**Authors:** Angela S. Choi, Madeline Theodorlis, Angelina Abbaticchio, Marisa Battistella

**Affiliations:** 1 Leslie Dan Faculty of Pharmacy, University of Toronto, Toronto, ON Canada; 2 Department of Pharmacy, Toronto General Hospital, University Health Network, Toronto, ON Canada

**Keywords:** Pharmacists, Prescriptive authority, Non-medical prescribing, Hemodialysis, Qualitative research

## Abstract

**Introduction:**

Pharmacist prescribing is expanding across care settings, supported by pharmacists’ expertise in pharmacology, therapeutics, disease management, and medication optimization. In settings where pharmacists can prescribe, patients and providers report positive outcomes. However, limited research has examined pharmacist prescribing in dialysis centers.

**Aim:**

This study explored patient and clinician perspectives on potential pharmacist prescribing in the outpatient hemodialysis unit at Toronto General Hospital, University Health Network (TGH-UHN) in Toronto, Canada.

**Method:**

Semi-structured, one-on-one interviews were conducted with English-speaking adults receiving hemodialysis, and clinicians, including nephrologists, pharmacists, dietitians, and nurse practitioners in the outpatient hemodialysis unit at TGH-UHN. Patient interviews focused on experiences receiving and filling prescriptions, interactions with pharmacists in the hemodialysis unit, and views on potential pharmacist prescribing in the unit. Clinician interviews explored the strengths and limitations of the current prescribing process in the hemodialysis unit, pharmacists’ role in the unit, perceived benefits and challenges of potential pharmacist prescribing, and strategies for implementation. Participants were recruited through convenience sampling until data saturation was reached. Interviews were audio-recorded, transcribed, and analyzed thematically using an inductive approach.

**Results:**

Eleven patients and 11 clinicians (six nephrologists, two pharmacists, two dietitians, and one nurse practitioner) were interviewed in June and July 2025. Reported challenges of the current prescribing process included communication gaps and delays in care, while accessibility of prescribers and interdisciplinary collaboration were identified as strengths. Pharmacists were recognized as valuable care team members for their expertise in medication management and rapport with patients. Anticipated benefits of pharmacist prescribing included improved medication optimization, workflow efficiency, timely care, and pharmaco-economic savings. Limited prescribing knowledge among some pharmacists was noted as a barrier. Implementation considerations included a collaborative approach, maintaining physician oversight, restricting prescribing to specific clinical areas, phased rollout, patient and clinician buy-in, adequate resources, and clearly defined roles and communication.

**Conclusion:**

Patients and clinicians were generally supportive of potential pharmacist prescribing in the hemodialysis unit, contingent on several considerations for implementation. Interviews with additional stakeholders in other dialysis care settings could further inform strategies for broader adoption.

**Supplementary Information:**

The online version contains supplementary material available at 10.1007/s11096-025-02084-x.

## Impact statements

- To maximize the patient, clinician, and system-level benefits of pharmacist prescribing, structured frameworks should be developed that clearly define scope of practice, support interdisciplinary communication, and ensure sufficient training and resources.
- This study underscores the value of collaborative prescribing models with physician oversight and suggests that expanding pharmacists’ roles may reduce physician workload while maintaining continuity of care.
- These findings provide a foundation for further research involving additional stakeholders to inform broader adoption of pharmacist prescribing in various dialysis settings.

## Introduction

In 2023, nearly 30,000 Canadians were receiving chronic dialysis [1], with more than 75% receiving hemodialysis (HD) in hospital-based settings. Patients receiving HD typically undergo four-hour treatments three times per week and take an average of 12 (± 5) medications daily [2–4]. These medications often require specific timing around dialysis sessions and frequent adjustments based on blood work results. Furthermore, patients receiving HD often interact with multiple healthcare services and providers. For example, a nephrologist may adjust medications for electrolyte balance, a family physician may prescribe antibiotics for an infection, and a pharmacist may provide patients with medication counseling.

Pharmacists are integral members of multidisciplinary HD care teams, providing evidence-based recommendations on medication selection, dosing, and therapeutic monitoring, often in collaboration with physicians and other prescribers, and in accordance with regulatory standards [5–7]. Pharmacists play a key role in addressing medication non-adherence, managing prescription renewals, and resolving duplicate or missing medications [5, 8, 9], all of which promote safer medication use by reducing inappropriate prescribing and optimizing therapy [10–19]. Pharmacist-led interventions, such as deprescribing, can significantly reduce pill burden and improve adherence without compromising patient safety [13–20]. Additionally, pharmacists support patient-centered care through counseling and motivational interviewing, which help reduce medication-related problems, improve satisfaction, and enhance overall patient outcomes.[21, 22, 23]

While prescribing is typically performed by physicians and nurse practitioners [7], pharmacists are increasingly recognized as qualified to prescribe given their expertise in pharmacology, therapeutics, disease management, pharmacokinetics, drug interactions, and adverse effects [24–26]. Many countries have extended prescribing authority to pharmacists to enhance patient access to medications [27, 28]. For example, the United Kingdom has implemented legislation providing pharmacists with independent prescribing authority [29]. Similarly, in Alberta, Canada, pharmacists in both community and hospital settings can independently adapt, renew, and initiate prescriptions, with additional prescribing authorization available [30, 31]. In other Canadian provinces, such as Ontario, community pharmacists are authorized to prescribe for minor ailments, such as uncomplicated urinary tract infections, gastroesophageal reflux disease, and conjunctivitis [32]. Evidence indicates that pharmacist prescribing is safe and effective [33]. In settings where pharmacists have prescribing rights, patients and healthcare providers report positive outcomes [34, 35], and a recent systematic review found that pharmacist prescribing services were both highly accessible and beneficial in improving access to medications [36]. Sharing prescribing responsibilities with pharmacists also reduces physicians’ workload, allowing them to focus on other complex health issues [33]. Furthermore, pharmacist prescribing may lower healthcare costs by optimizing therapy and preventing hospital readmissions [37, 38].

Despite growing evidence that pharmacists possess the competencies to prescribe safely, there are several challenges to implementing this practice [33, 39]. These include concerns about liability, inter-provider communication, and inadequate infrastructure, such as limited time, staffing, training resources, and technology [8, 34, 40, 41]. Addressing these barriers requires coordinated efforts, including policy changes and organizational supports [33]. Given the complexities of medication management for patients receiving HD, multidisciplinary teams with clear communication pathways are essential to ensure safe and effective pharmacist prescribing [33, 39, 42]. Ongoing education and training for pharmacists and other healthcare providers are also critical for successful integration of pharmacists into prescribing models in HD settings [8, 34, 40, 41]. To date, no study has explored patient and clinician perspectives on potential pharmacist prescribing in outpatient HD centers [42–44], and research is needed to identify barriers and facilitators for its implementation.

### Aim

This study explored patient and clinician perspectives on potential pharmacist prescribing in an outpatient HD unit in Toronto, Canada.

## Method

### Study design

This study involved one-on-one, semi-structured interviews and adopted a qualitative descriptive design, providing rich, detailed data that accurately reflects participants’ perspectives [45]. Findings are reported in accordance with the Consolidated Criteria for Reporting Qualitative Research (COREQ) guidelines [46].

### Setting

This single-center study was conducted in the outpatient HD unit at Toronto General Hospital, University Health Network (TGH-UHN), in Toronto, Ontario, Canada. The research team comprised the Principal Investigator (MB, female), a pharmacist clinician scientist; two pharmacy research coordinators (MT, AA, both female) with a Master of Public Health; and a pharmacy research student (AC, female). MT and AA, experienced in qualitative interviewing and analysis, provided training and oversight to AC.

At TGH-UHN, prescribing for patients on HD typically involves nephrologists and nurse practitioners in the HD unit, and family physicians in the community. The HD team also comprises dietitians and social workers, as well as a full-time pharmacist responsible for comprehensive medication management, including consulting with and educating the clinical team on appropriate medication use and dosing.

### Sampling and recruitment

Participants were recruited between June and July 2025. Eligible patients were aged 18 years or older, receiving outpatient HD at TGH-UHN, English-speaking, and without cognitive impairment. Patients were approached by AC and MT in person during their dialysis session and provided with a brief verbal overview of the study. Eligible clinicians included nephrologists, pharmacists, nurse practitioners, and dietitians working in the same unit. AC and MT contacted clinicians by email with a study description and instructions to contact the team if they were interested in participating. Patient and clinician recruitment continued until data saturation was achieved. All participants provided written informed consent.

### Interview guide development

Semi-structured interview guides (Online Resources 1 and 2) were developed by the study coordinators (MT, AA), the pharmacy research student (AC), and the Principal Investigator (MB), with input from a pharmacist, nurse practitioner, and social worker in the HD unit. The guides included open-ended questions exploring the strengths and challenges of the current prescribing process for patients on HD, the perceived role of pharmacists in the HD unit, and perspectives on potential pharmacist prescribing in the unit. Probing questions were incorporated to capture a comprehensive understanding of participant views. The guides were pilot tested with one social worker from the outpatient HD unit and one HD patient partner at TGH-UHN, then revised for clarity.

### Data collection

Interviews were conducted by AC between June and July 2025. Patient interviews occurred in person at the bedside during dialysis sessions. Clinician interviews were conducted either in person or via Microsoft Teams. There was no prior relationship between AC and the study participants. The first three patient and clinician interviews were supervised by MT, who provided AC with feedback on interviewing technique. Interviews were audio-recorded and transcribed verbatim. Transcripts were verified against recordings for accuracy, and audio files were subsequently deleted. Identifying information was removed to ensure confidentiality. Due to time and resource constraints, transcripts were not returned to participants for comment or correction.

Patient demographic data were obtained from electronic medical records (EMR) or directly from the patient when unavailable in their EMR. Variables included age, sex, ethnicity, education, marital status, comorbidities (diabetes, hypertension, cardiovascular disease, dyslipidemia), total number of medications, and dialysis vintage. Clinician demographic data were self-reported and included age, sex, ethnicity, clinical role, and years of clinical practice.

### Data analysis

Patient and clinician characteristics were summarized using descriptive statistics. Categorical variables were reported as frequencies and percentages, and continuous variables as means with standard deviations, or medians with interquartile ranges. Transcripts were analyzed manually using the “comments” and “track changes” features in Microsoft Word. Analysis followed an iterative, inductive approach, whereby themes and subthemes were derived “directly” from the interview data [47].

To ensure inter-rater reliability, AC, MT, and AA independently coded the first three patient and clinician transcripts to develop a preliminary coding framework. This framework was then applied to all subsequent transcripts, which were independently coded by the same researchers. The team met regularly to address discrepancies and refine the framework. Further, AC maintained a reflexive journal throughout the interview process, where she took notes before and after each interview to consider how her personal and professional bias might influence data collection and analysis. AC, MT, and AA openly discussed assumptions or biases during analysis meetings. Interviews and analysis occurred concurrently until thematic saturation was reached, and no new themes or subthemes emerged. Saturation was assessed separately for patients and clinicians and was determined through real-time coding and team consensus.

Themes and subthemes were mapped to the Theoretical Domain Framework (TDF), a validated framework developed by psychology theorists and implementation researchers to guide the design of implementation interventions [48, 49]. The TDF includes 14 domains: knowledge; skills; social or professional role and identity; beliefs about capabilities; optimism; beliefs about consequences; reinforcement; intentions; goals; memory, attention and decision processes; environmental context and resources; social influences; emotion; and behavioural regulation [49]. AC conducted the initial mapping, which was then reviewed by MT and AA, with remaining discrepancies resolved by MB. Due to time and resource constraints, participants did not review the findings.

## Ethics approval

The study received approval from the University Health Network Research Ethics Board (Study ID: 25–5303) on February 1, 2025.

## Results

Of 240 patients in the HD unit at TGH-UHN, 127 were eligible to participate. Of these, 19 were approached and 11 consented and interviewed. All 18 eligible clinicians were contacted with 11 agreeing to participate (six nephrologists, two pharmacists, two dietitians, and one nurse practitioner) (Table 1). Interviews lasted 11 to 26 minutes. Patients were a mean age of 66 years, and more than half (55%) were white. Clinicians reported they had between three and 36 years of clinical practice experience.

**Table 1:** Participant interview characteristics

Characteristic	Patients (N = 11)	Clinicians (N = 11)
*Mean age *(± *SD*)	66 (± 14)	45 (± 12) *
*Sex *(%)		
Male	5 (45)	5 (45)
Female	6 (55)	6 (55)
*Ethnicity *(%)		
Black, African	1 (9)	1 (9)
Black, Caribbean	1 (9)	0
East Asian	0	1 (9)
South Asian	1 (9)	1 (9)
Southeast Asian	2 (18)	4 (36)
White	6 (55)	4 (36)
*Clinical role *(%)		
Dietitian	–	2 (18)
Nephrologist	–	6 (55)
Nurse practitioner	–	1 (9)
Pharmacist	–	2 (18)
*Highest level of education *(%)		
Elementary school (up to grade 6)	1 (9)	–
Middle school (up to grade 8)	0	–
High school (up to grade 13)	2 (18)	–
College/university	7 (64)	–
Postgraduate	1 (9)	–
*Marital status*		
Single	3 (27)	–
Divorced	1 (9)	–
Married/common law	5 (45)	–
Widowed	2 (18)	–
*Comorbidities *(%)		
Diabetes	7 (64)	–
Hypertension	8 (82)	–
Cardiovascular disease	6 (55)	–
Lipid disorder	6 (55)	–
Median years of clinical practice (IQR)		12 (10–19) *
Total number of medications (± SD)	11 (± 5)	–
Median dialysis vintage, in months (IQR)	32 (10–48)	–
Median dialysis vintage at TGH-UHN, in months (IQR)	27 (10–40)	–

*SD* standard deviation, *IQR* interquartile range, *TGH-UHN* Toronto General Hospital – University Health Network

^*^n = 10; one clinician preferred not to answer

From patient and clinician interviews, four main themes and several subthemes were identified and mapped to nine TDF domains (Table 2). All coded interview data are available upon reasonable request.

**Table 2:** Themes identified by patients and clinicians mapped to the Theoretical Domain Framework (TDF)

Theme	Subtheme	Patients	Clinicians	TDF domains [49]
Strengths and challenges of the current prescribing process	Prescribers are accessible	✓		Environmental context and resources
	Communication gaps lead to delays	✓		
	Strong interdisciplinary collaboration		✓	
	Electronic medical record system		✓	
	Multiple prescribers challenge communication		✓	
Perceived role of pharmacists in the hemodialysis unit	Optimize medication management and educate patients	✓	✓	Social or professional role and identity
	Accessible members of the care team	✓		
	Assist with administrative tasks of prescribing		✓	
Perceived benefits and barriers of pharmacist prescribing	Improve workflow efficiency and timely care	✓	✓	Beliefs about consequences
	Support medication adherence and safety		✓	Knowledge; Skills
	Pharmaco-economic savings		✓	Beliefs about consequences
	Pharmacists have existing rapport with patients	✓	✓	Skills; Beliefs about capabilities
	Pharmacists’ skillset and expertise	✓	✓	Knowledge; Skills; Beliefs about capabilities
Implementation considerations for pharmacist prescribing	Collaborative prescribing	✓	✓	Goals
	Emphasis on physician oversight	✓	✓	Social influences
	Support for prescribing in specific clinical areas	✓	✓	Behavioural regulation
	Phased approach to implementation		✓	
	Patient and clinician buy-in		✓	Social influences
	Resources to support increased responsibility of pharmacists		✓	Environmental context and resources
	Clearly defined responsibilities and communication among the hemodialysis care team		✓	
	Inform patients of changes (e.g., verbal, written, digital communication)	✓	✓	Goals

### Theme 1: Strengths and challenges of the current prescribing process

Patients and clinicians identified several environmental context- and resource*-*related [49] strengths and challenges in current prescribing practices for patients on HD. Many patients reported that prescribers in the HD unit are readily accessible and that requesting prescriptions is generally straightforward. Patients and clinicians highlighted the convenience of electronic faxing to send prescriptions directly to community pharmacies. However, some patients described delays in receiving medications.

*If one of the several medications doesn’t have a refill, the pharmacist will have to get a fax from the doctor or nurse requesting additional refill… as a patient, I find it frustrating.* (Patient 11, female, age 69)

Several clinicians emphasized interdisciplinary collaboration among the HD care team as a strength. However, the involvement of multiple or rotating prescribers in the HD unit can lead to miscommunication, lack of follow-up, and delays in care.

*The follow-up or communication, that’s where you sometimes see the gaps, where something has been adjusted and maybe not fully communicated with the team or [there’s] not an opportunity because they’ve been moved to a different area, different rotation.* (Clinician 01, dietitian)

Finally, many clinicians reported that the hospital’s EMR system supports prescribing through its convenience and record-keeping functions, though some mentioned its limitations, including difficulty navigating the system or inaccuracies in medication lists.

### Theme 2: Perceived role of pharmacists in the hemodialysis unit

Participants described pharmacists as integral members of the HD care team, valued for their social or professional roles and identities [49] within the unit. Several nephrologists, one nurse practitioner, and multiple patients characterized HD pharmacists as medication experts who optimize therapeutic regimens and resolve complex clinical issues, such as identifying missing medications or adjusting dosages to prevent complications. One pharmacist detailed their responsibilities as:

*Reviewing the appropriateness, the dosing frequency, checking for interactions, reviewing blood work and performing assessments and recommendations for any drug therapy issues… Also providing any education to the patients and then collaborating with other healthcare team members.* (Clinician 10, pharmacist)

Both patients and clinicians emphasized pharmacists’ role in patient education, including explaining medication changes and potential side effects:

*[Explaining what] the purpose is of something being increased or decreased and how it’s going to affect me, or if a new prescription is ordered, to find out if there are chances that I would have some reaction to it and how to deal with whatever the problem is.* (Patient 08, female, age 81)

Patients also highlighted pharmacists’ accessibility and approachability, noting their availability for structured counseling or to address medication-related concerns upon request: “*[they’re] always available to answer my questions… they are an integral part of my treatment… I consider them part of a team in my well-being”.* (Patient 11, female, age 69).

In additional to clinical and educational roles, nephrologists noted pharmacists’ involvement in administrative activities, such as liaising with community pharmacies and assisting patients with medication access and coverage programs.

### Theme 3: Perceived benefits and barriers of pharmacist prescribing

Patients and clinicians identified several potential benefits and one perceived barrier related to pharmacists’ capabilities, knowledge, and skills [49]. Most participants expressed confidence in pharmacists’ ability to prescribe, citing their expertise and established rapport with patients. Patients described pharmacists in the HD unit as “*accessible, friendly, knowledgeable, caring, and articulate”* (Patient 11, female, age 69). Most clinicians echoed these views, and one nephrologist noted that granting pharmacists prescribing authority would be *“taking advantage of a skillset that they have”* (Clinician 06, nephrologist). Many clinicians believed that pharmacists’ existing relationships with patients and familiarity with their medical histories could support continuity of care. Several nephrologists also highlighted pharmacists’ expertise in adherence strategies and patient education, suggesting that pharmacist prescribing could improve patient safety by optimizing medication regimens.

Both patients and clinicians indicated that pharmacist prescribing could reduce physicians’ workload, allowing them to focus on more complex care needs:

*The more care you have, including pharmacists that can help you, if it’s to prescribe even minor medications to help… the better… you’re taking some of the work off the doctors, so you’re helping everybody in general.* (Patient 09, male, age 50)

Additionally, several nephrologists and both dietitians noted that pharmacist prescribing could streamline processes for patients without a family physician or those requesting prescriptions during dialysis sessions:

*[Patients] don’t often see their family doctor, or maybe they don’t have a family doctor, they do usually ask for prescriptions for minor ailments when they’re here. So [pharmacist prescribing] would help with the efficiency… patients don’t have to wait to see the physician because they might be able to see a pharmacist sooner… a lot of the physicians, they do just take the pharmacist’s recommendation anyway, so it would kind of just be cutting out the middleman.* (Clinician 04, dietitian)

A key barrier noted by one nephrologist was variability in pharmacists’ knowledge and capabilities:

*There are some pharmacists who I would trust implicitly… some pharmacists have an incredibly high knowledge base… there are other pharmacists I would not wish them to be involved with any prescribing… I will double check things just because I feel that there is a significant knowledge gap and a lack of familiarity with the clinical scenarios.* (Clinician 08, nephrologist)

Finally, one nephrologist and one pharmacist suggested that pharmacists’ knowledge of medication cost and coverage, combined with their role in medication optimization, may contribute to pharmaco-economic savings.

### Theme 4: Implementation considerations for pharmacist prescribing.

Participants identified several key considerations for implementing pharmacist prescribing in the HD unit, emphasizing changes in behaviour and responsibilities [49] among the HD clinical team and the need for additional resources. A few patients and clinicians preferred a team-based prescribing approach to ensure *“more eyes on it than just one person”* (Patient 06, female, age 37) and to maintain consistent communication across the HD team with prescribing decisions that are *“endorsed or vetted by everybody else within the clinical team”* (Clinician 03, nurse practitioner). Many patients, a nephrologist, and a pharmacist emphasized the need for continued physician oversight:

*If the pharmacist examine you and… prescribe you… if they do under supervision of a specialist, then I think nobody have any issues… if the team is involved, because the doctor actually is our main person who is in charge… under his wing, and he’s aware about it… why not?* (Patient 01, male, age 53)

*I think the medical practitioner should still be the primary prescriber, but giving pharmacists the ability to prescribe if needed.* (Clinician 10, pharmacist)

Many clinicians, and some patients, suggested starting with limited prescribing responsibilities, such as prescription refills, minor ailments (e.g., gastroesophageal reflux disease), or specific clinical areas like anticoagulation, antihypertensives, or calcium-phosphate management. A phased approach with gradual expansion of responsibilities, accompanied by frequent review and scope adjustments, was widely recommended by clinicians, particularly nephrologists. Most participants, especially clinicians, emphasized the importance of a well-defined scope of practice, clear guidelines, and strong communication processes among the HD care team. Clinicians also noted the need for adequate pharmacy staffing and remuneration to *“support pharmacists playing a bigger role on the dialysis unit”* (Clinician 02, pharmacist).

Clinicians emphasized the importance of clinician and patient buy-in *“to ensure that patients would be comfortable in receiving direct prescription from the pharmacist involved in their care”* (Clinician 11, nephrologist). Lastly, all participants suggested informing patients of changes verbally at the bedside, or through written or digital communication (e.g., newsletters, online patient portals), with some recommending reiterations during monthly rounds.

## Discussion

This study explored patient and clinician perspectives on pharmacist prescribing in the outpatient HD unit at TGH-UHN in Toronto, Canada. Patients and clinicians highlighted prescriber accessibility and interdisciplinary collaboration as strengths of the current prescribing model. Challenges included delayed care resulting from communication gaps among multiple prescribers, and inaccuracies in medication lists within the hospital EMR. Participants emphasized that pharmacists play a vital role in HD care through their expertise in medication management, patient education, and established rapport with patients. Anticipated benefits of pharmacist prescribing included improved medication adherence and safety, enhanced workflow efficiency, faster access to care, and cost savings. A potential barrier noted by one nephrologist is that some pharmacists may lack the knowledge or skills required to prescribe. Participants supported a collaborative model in which pharmacists prescribe under physician oversight. Clinicians further recommended a phased rollout with gradual expansion of responsibilities, clear roles and communication among the healthcare team, adequate resources, and clinician and patient buy-in. All participants suggested transparent communication with patients regarding changes in prescribing practices.

Our findings contribute to broader literature on pharmacist prescribing in community and hospital-based settings [50–52] and offer novel insights by capturing patient and clinician perspectives on potential pharmacist prescribing within an outpatient HD unit, a setting that remains underexplored. Consistent with existing literature [21–26], participants viewed pharmacists as essential members of multidisciplinary HD care teams due to their expertise in medication management and their role in patient‑centered medication counseling. Our findings further support the notion that pharmacists currently contribute to prescribing practices through medication selection and dosing suggestions, despite lacking formal prescribing authority [53]. Furthermore, while the HD team was generally viewed as collaborative, participants noted that the current prescribing model in the unit is sometimes challenged by inconsistent communication among the many prescribers involved in patients’ care. Such communication barriers are well-recognized in HD care, where patients often present with complex, multi-faceted and time-sensitive needs, and rely on several prescribers across various care settings [54]. Fragmented communication and medication documentation issues pose risks to continuity of care and patient safety [55].

Perceived benefits of pharmacist prescribing identified in this study are consistent with prior research demonstrating high patient satisfaction with pharmacist counseling, improved quality of life [28, 56, 57], fewer medication-related problems, and improved clinical outcomes, often achieved through collaboration with nephrologists or the HD team [8, 58]. Improved patient care and reduced physician workload [28, 29, 50, 56], were also reflected in our findings. Participants emphasized that the potential value of pharmacist prescribing also includes improved workflow efficiency and timely access to medications, considerations particularly relevant in HD settings where delays in care can have significant clinical consequences [54]. However, expanding pharmacists’ responsibilities would require additional resources to ensure they can continue to focus on critical aspects of patient care. This aligns with previous research highlighting financial and human resource related barriers [33, 38, 43] and the need for appropriate remuneration, staffing, and workload considerations to support pharmacists’ expanded roles [50, 52, 58].

These findings provide several implications for policy and practice. First, support for pharmacist prescribing among participants was contingent on a collaborative prescribing model with physician oversight, indicating that there may be limited support within this setting for independent pharmacist prescribing. Concerns raised regarding pharmacists’ prescribing competencies is an issue consistent with previously reported barriers, including discomfort with increased responsibility, resource constraints, and training and accreditation requirements [27, 29, 40, 50, 51, 57, 59]. These findings highlight the importance of investing in training programs to equip all pharmacists with the competencies to undertake prescribing responsibilities. Second, given communication challenges already present within the HD unit, introducing an additional prescriber may exacerbate existing gaps and create delays in patient care. As noted by participants in our study, introducing pharmacist prescribing requires clear communication across the HD care team. Thus, implementation efforts should include strategies to enhance communication practices in the HD unit, such as optimizing EMR systems and reinforcing comprehensive medication documentation practices. Furthermore, clinicians envisioned pharmacist prescribing as fitting within existing workflows, including medication reviews and ongoing medication optimization [60, 61], suggesting readiness for the adoption of pharmacist prescribing in HD units, provided that clear roles and communication pathways are established. Finally, while pharmacist prescribing has the potential to reduce physician workload and improve patient access to timely care, it will require adequate financial compensation and staffing. Policies similar to those adopted in the United Kingdom, such as integrating prescribing competencies earlier in pharmacy education, may help address these concerns and support implementation [33].

To our knowledge, this is the first study to explore both patient and clinician perspectives on pharmacist prescribing in outpatient HD units through qualitative interviews [39–41]. Our findings offer insight into specific facilitators, barriers, and considerations for implementation of pharmacist prescribing in an HD setting. Convenience sampling was employed to maximize recruitment among over 120 eligible patients in the outpatient HD unit at TGH-UHN. To enhance rigour and ensure high inter-rater reliability, interview transcripts underwent independent review by multiple research team members, followed by regular discussions to develop consensus. The application of the TDF, a widely recognized tool for identifying factors that influence policy implementation and inform intervention design [48, 49], further strengthened our methodology and supports the development of actionable strategies to implement pharmacist prescribing in HD settings.

Some limitations should be noted. Patients who did not speak English, with cognitive impairment, and on the evening or nocturnal HD shifts were excluded. This exclusion may have overestimated patients’ openness pharmacist prescribing in the HD unit, as language barriers and cognitive impairment can influence patients’ experiences with care [62–65], and patients on later dialysis shifts have fewer interactions with pharmacists. Future research using interpreters or language translation services, and interviewing patients from all HD shifts, could better capture the perspectives of the broader HD population. Finally, this study was conducted at a single HD unit in an urban, academic hospital, and pharmacist involvement may differ across HD settings. Future research should involve diverse patient populations and geographically varied HD centers, including rural and remote areas, to capture a wider range of perspectives.

## Conclusion

This study explored patient and clinician perspectives on potential pharmacist prescribing in an outpatient HD unit, identifying key benefits, challenges, and considerations for expanding pharmacists’ roles in this setting. Patients and clinicians expressed support for a collaborative pharmacist prescribing model within the outpatient HD unit, while highlighting the importance of clear communication processes, well-defined roles, and adequate training and resources for safe and effective implementation. These findings provide a foundation for future research involving additional stakeholders to inform broader adoption of pharmacist prescribing across dialysis settings, ultimately improving patient care, and enhancing healthcare delivery for the HD population.

## Supplementary Information

Below is the link to the electronic supplementary material.

Supplementary file1 (PDF 226 KB)

Supplementary file2 (PDF 200 KB)

## Data Availability

Full data are available from the corresponding author on reasonable request.

## References

[1] Annual statistics on organ replacement in Canada, 2012 to 2021. Canadian Institute for Health Information. 2023. https://www.cihi.ca/en/annual-statistics-on-organ-replacement-in-canada-2012-to-2021. Accessed 26 Mar 2025.

[2] Chiu Y-W, Teitelbaum I, Misra M, et al. Pill burden, adherence, hyperphosphatemia, and quality of life in maintenance dialysis patients. Clin J Am Soc Nephrol. 2009. 10.2215/CJN.00290109. 19423571 10.2215/CJN.00290109 PMC2689877

[3] Battistella M, Fleites R, Wong R, et al. Development, validation, and implementation of a medication adherence survey to seek a better understanding of the hemodialysis patient. Clin Nephrol. 2016. 10.5414/CN108654. 26636327 10.5414/CN108654

[4] Manley HJ, McClaran ML, Overbay DK, et al. Factors associated with medication-related problems in ambulatory hemodialysis patients. Am J Kidney Dis. 2003. 10.1053/ajkd.2003.50048. 12552501 10.1053/ajkd.2003.50048

[5] Tang I, Vrahnos D, Hatoum H, et al. Effectiveness of clinical pharmacist interventions in a hemodialysis unit. Clin Ther. 1993;15:456–64. 8519051

[6] Qudah B, Albsoul-Younes A, Alawa E, et al. Role of clinical pharmacist in the management of blood pressure in dialysis patients. Int J Clin Pharm. 2016. 10.1007/s11096-016-0317-2. 27194097 10.1007/s11096-016-0317-2

[7] Regulated health professions. Government of Ontario. 2023. https://www.ontario.ca/page/regulated-health-professions. Accessed 28 Aug 2025

[8] Daifi C, Feldpausch B, Roa P-A, et al. Implementation of a clinical pharmacist in a hemodialysis facility: a quality improvement report. Kidney Med. 2021. 10.1016/j.xkme.2020.11.015. 33851119 10.1016/j.xkme.2020.11.015 PMC8039404

[9] Thwin O, Han M, Tao X, et al. Feasibility study of wrist-based wearable activity tracker in hemodialysis patients. J Am Soc Nephrol. 2021. 10.1681/asn.20203110s1392a.34281959

[10] Ogilvie M, Nissen L, Kyle G, et al. An evaluation of a collaborative pharmacist prescribing model compared to the usual medical prescribing model in the emergency department. Res Social Adm Pharm. 2022. 10.1016/j.sapharm.2022.05.005. 35581126 10.1016/j.sapharm.2022.05.005

[11] Bondurant-David K, Dang S, Levy S, et al. Issues with deprescribing in haemodialysis: a qualitative study of patient and provider experiences. Int J Pharm Pract. 2020. 10.1111/ijpp.12674. 33094884 10.1111/ijpp.12674

[12] Linsky A, Zimmerman KM. Provider and system-level barriers to deprescribing: interconnected problems and solutions. Public Policy Aging Report. 2018. 10.1093/ppar/pry030.

[13] Gerardi S, Sperlea D, Levy SOL, et al. Implementation of targeted deprescribing of potentially inappropriate medications in patients on hemodialysis. Am J Health Syst Pharm. 2022. 10.1093/ajhp/zxac190. 35881917 10.1093/ajhp/zxac190

[14] Trenaman SC, Kennie-Kaulbach N, d’Entremont-MacVicar E, et al. Implementation of pharmacist-led deprescribing in collaborative primary care settings. Int J Clin Pharm. 2022. 10.1007/s11096-022-01449-w. 35794285 10.1007/s11096-022-01449-w PMC9261167

[15] Kassis A, Moles R, Carter S. Stakeholders’ perspectives and experiences of the pharmacist’s role in deprescribing in ambulatory care: a qualitative meta-synthesis. Res Social Adm Pharm. 2024. 10.1016/j.sapharm.2024.04.014. 38685144 10.1016/j.sapharm.2024.04.014

[16] Kose E, Endo H, Hori H, et al. Association of pharmacist-led deprescribing intervention with the functional recovery in convalescent setting. Pharmazie. 2022. 10.1691/ph.2022.2323. 35655381 10.1691/ph.2022.2323

[17] Chan M, Plakogiannis R, Stefanidis A, et al. Pharmacist-led deprescribing for patients with polypharmacy and chronic disease states: a retrospective cohort study. J Pharm Pract. 2023. 10.1177/08971900221097246. 35522029 10.1177/08971900221097246

[18] Elbeddini A, Zhang CXY. The pharmacist’s role in successful deprescribing through hospital medication reconciliation. Can Pharm J/Revue des Pharmaciens du Canada. 2019. 10.1177/1715163519836136. 10.1177/1715163519836136 PMC6512178 31156730

[19] Lui E, Wintemute K, Muraca M, et al. Pharmacist-led sedative-hypnotic deprescribing in team-based primary care practice. Can Pharm J/Revue des Pharmaciens du Canada. 2021. 10.1177/17151635211014918. 10.1177/17151635211014918 PMC8282922 34345321

[20] Falah MJ, Jasim AL. The impact of implementing a pharmacist-led deprescribing program on medication adherence among hemodialysis patients. Al-Rafidain J Med Sci. 2023. 10.54133/ajms.v5i1S.290.

[21] Kuntz JL, Safford MM, Singh JA, et al. Patient-centered interventions to improve medication management and adherence: a qualitative review of research findings. Patient Educ Couns. 2014. 10.1016/j.pec.2014.08.021. 25264309 10.1016/j.pec.2014.08.021 PMC5830099

[22] Okumura LM, Rotta I, Correr CJ. Assessment of pharmacist-led patient counseling in randomized controlled trials: a systematic review. Int J Clin Pharm. 2014. 10.1007/s11096-014-9982-1. 25052621 10.1007/s11096-014-9982-1

[23] Tadesse YB, Sendekie AK, Mekonnen BA, et al. Pharmacists’ medication counseling practices and knowledge and satisfaction of patients with an outpatient hospital pharmacy service. Inquiry. 2023. 10.1177/00469580231219457. 38131171 10.1177/00469580231219457 PMC10748552

[24] Raiche T, Pammett R, Dattani S, et al. Community pharmacists’ evolving role in Canadian primary health care: a vision of harmonization in a patchwork system. Pharm Pract (Granada). 2020. 10.18549/PharmPract.2020.4.2171. 33149795 10.18549/PharmPract.2020.4.2171 PMC7603659

[25] Aghili M, Kasturirangan MN. Management of drug–drug interactions among critically ill patients with chronic kidney disease: impact of clinical pharmacist’s interventions. Indian J Crit Care Med. 2021. 10.5005/jp-journals-10071-23919. 34866818 10.5005/jp-journals-10071-23919 PMC8608633

[26] Omboni S, Caserini M. Effectiveness of pharmacist’s intervention in the management of cardiovascular diseases. Open Heart. 2018. 10.1136/openhrt-2017-000687. 29344376 10.1136/openhrt-2017-000687 PMC5761304

[27] Stewart D, Jebara T, Cunningham S, et al. Future perspectives on nonmedical prescribing. Ther Adv Drug Saf. 2017. 10.1177/2042098617693546. 28607668 10.1177/2042098617693546 PMC5455843

[28] Jebara T, Cunningham S, Maclure K, et al. Key stakeholders’ views on the potential implementation of pharmacist prescribing: a qualitative investigation. Res Social Adm Pharm. 2020. 10.1016/j.sapharm.2019.06.009. 31253499 10.1016/j.sapharm.2019.06.009

[29] Jebara T, Cunningham S, Maclure K, et al. Stakeholders’ views and experiences of pharmacist prescribing: a systematic review. Br J Clin Pharmacol. 2018. 10.1111/bcp.13624. 29873098 10.1111/bcp.13624 PMC6089831

[30] Yuksel N, Eberhart G, Bungard TJ. Prescribing by pharmacists in Alberta. Am J Health-Syst Pharm. 2008. 10.2146/ajhp080247. 18997141 10.2146/ajhp080247

[31] Prescribing authority of pharmacists across Canada. Canadian Pharmacists Association. 2025. https://www.pharmacists.ca/cpha-ca/assets/File/pharmacy-in-canada/PharmacistPrescribingAuthority_EN_web.pdf. Accessed 26 Mar 2025.

[32] Ontario pharmacists now authorized to prescribe for minor ailments. Ontario College of Pharmacists. 2023. https://www.ocpinfo.com/ontario-pharmacists-now-authorized-to-prescribe-for-minor-ailments/. Accessed 26 Mar 2025.

[33] Al Raiisi F, Cunningham S, Stewart D. A qualitative, theory-based exploration of facilitators and barriers for implementation of pharmacist prescribing in chronic kidney disease. Int J Clin Pharm. 2024. 10.1007/s11096-024-01794-y. 39230784 10.1007/s11096-024-01794-y PMC11576801

[34] Majercak KR. Advancing pharmacist prescribing privileges: is it time? J Am Pharm Assoc. 2019. 10.1016/j.japh.2019.08.004. 10.1016/j.japh.2019.08.004 31521588

[35] Ryan KL, Jakeman B, Conklin J, et al. Treatment of patients with HIV or hepatitis C by pharmacist clinicians in a patient-centered medical home. Am J Health-Syst Pharm. 2019. 10.1093/ajhp/zxz059. 31053839 10.1093/ajhp/zxz059

[36] Walpola RL, Issakhany D, Gisev N, et al. The accessibility of pharmacist prescribing and impacts on medicines access: a systematic review. Res Social Adm Pharm. 2024. 10.1016/j.sapharm.2024.01.006. 38326207 10.1016/j.sapharm.2024.01.006

[37] Hawes EM, Misita C, Burkhart JI, et al. Prescribing pharmacists in the ambulatory care setting: experience at the University of North Carolina Medical Center. Am J Health-Syst Pharm. 2016. 10.2146/ajhp150771. 27605321 10.2146/ajhp150771

[38] Law MR, Morgan SG, Majumdar SR, et al. Effects of prescription adaptation by pharmacists. BMC Health Serv Res. 2010. 10.1186/1472-6963-10-313. 21083922 10.1186/1472-6963-10-313 PMC2994856

[39] Al Raiisi F, Stewart D, Ashley C, et al. A theoretically based cross-sectional survey on the behaviors and experiences of clinical pharmacists caring for patients with chronic kidney disease. Res Social Adm Pharm. 2021. 10.1016/j.sapharm.2020.05.005. 10.1016/j.sapharm.2020.05.005 32534956

[40] Emmerton L, Marriott J, Bessell T, et al. Pharmacists and prescribing rights: review of international developments. J Pharm Pharm Sci. 2005;8:217–25. 16124933

[41] Al-Abdelmuhsin L, Al-Ammari M, Babelghaith SD, et al. Assessment of pharmacists’ knowledge and practices towards prescribed medications for dialysis patients at a tertiary hospital in Riyadh Saudi Arabia. Healthcare. 2021. 10.3390/healthcare9091098. 34574871 10.3390/healthcare9091098 PMC8468859

[42] Ardavani A, Curtis F, Hopwood E, et al. Effect of pharmacist interventions in chronic kidney disease: a meta-analysis. Nephrol Dial Transplant. 2024. 10.1093/ndt/gfae221. 10.1093/ndt/gfae221 PMC12209859 39384574

[43] Al Raiisi F, Stewart D, Fernandez‐Llimos F, et al. Clinical pharmacy practice in the care of chronic kidney disease patients: a systematic review. Int J Clin Pharm. 2019. 10.1007/s11096-019-00816-4. 30963447 10.1007/s11096-019-00816-4 PMC6554252

[44] Livori R, Shaji C, Tran DGN, et al. Renal clinical pharmacy services and outcomes for patients on dialysis: a scoping review. J Pharm Pract Res. 2025. 10.1002/jppr.70006.

[45] Lambert VA, Lambert CE. Editorial: qualitative descriptive research: an acceptable design. Pac Rim Int J Nurs Res Thail. 2012;16:255–6.

[46] Tong A, Sainsbury P, Craig J. Consolidated criteria for reporting qualitative research (COREQ): a 32-item checklist for interviews and focus groups. Int J Qual Health Care. 2007. 10.1093/intqhc/mzm042. 17872937 10.1093/intqhc/mzm042

[47] Thomas DR. A general inductive approach for analyzing qualitative evaluation data. Am J Eval. 2006. 10.1177/1098214005283748.

[48] Islam R, Tinmouth AT, Francis JJ, et al. A cross-country comparison of intensive care physicians’ beliefs about their transfusion behaviour: a qualitative study using the theoretical domains framework. Implement Sci. 2012. 10.1186/1748-5908-7-93. 22999460 10.1186/1748-5908-7-93 PMC3527303

[49] Murphy K, O’Connor D. Understanding diagnosis and management of dementia and guideline implementation in general practice: a qualitative study using the theoretical domains framework. Implement Sci. 2014. 10.1186/1748-5908-9-31. 24581339 10.1186/1748-5908-9-31 PMC4015883

[50] Almawed R, Shiu J, Bungard T, et al. Pharmacist prescribing at inpatient discharge in Alberta. Can J Hosp Pharm. 2023. 10.4212/cjhp.3346. 37767376 10.4212/cjhp.3346 PMC10522341

[51] Van Berlo-Van De Laar IRF, Sluiter HE, Riet E, et al. Pharmacist-led medication reviews in pre-dialysis and dialysis patients. Res Social Adm Pharm. 2020. 10.1016/j.sapharm.2020.02.006. 32111533 10.1016/j.sapharm.2020.02.006

[52] Heck T, Gunther M, Bresee L, et al. Independent prescribing by hospital pharmacists: patterns and practices in a Canadian province. Am J Health Syst Pharm. 2015. 10.2146/ajhp150080. 26637516 10.2146/ajhp150080

[53] Vuong V, Bhojwani R, Sengar A, et al. Prescription modification by pharmacists in a hospital setting: are Ontario pharmacists ready? CJHP. 2021. 10.4212/cjhp.v74i3.3151. 34248164 10.4212/cjhp.v74i3.3151 PMC8237955

[54] Zhang T, Mohsen M, Abbaticchio A, et al. Patients’ experiences of medication management while navigating ongoing care between outpatient services: a qualitative case study of patients on hemodialysis. Explor Res Clin Soc Pharm. 2024. 10.1016/j.rcsop.2024.100418. 38374965 10.1016/j.rcsop.2024.100418 PMC10875289

[55] Talson MD, Ferreira Da Silva P, Finlay J, et al. Patient, caregiver, and provider perspectives on improving provider-patient interactions in hemodialysis: a qualitative study. Can J Kidney Health Dis. 2025. 10.1177/20543581241309986. 39758856 10.1177/20543581241309986 PMC11696963

[56] Brennan B, Strawbridge J, Stewart D, et al. An umbrella review of pharmacist prescribing: stakeholders’ views and impact on patient outcomes. Int J Pharm Pract. 2024. 10.1093/ijpp/riae013.009.

[57] Gray M, Mysak T. The road to pharmacist prescribing in Alberta Health Services. Am J Health Syst Pharm. 2016. 10.2146/ajhp150786. 27605324 10.2146/ajhp150786

[58] Makowsky M, Guirguis L, Hughes C, et al. Factors influencing pharmacists’ adoption of prescribing: qualitative application of the diffusion of innovations theory. Implement Sci. 2013. 10.1186/1748-5908-8-109. 24034176 10.1186/1748-5908-8-109 PMC3847669

[59] Zhou M, Desborough J, Parkinson A, et al. Barriers to pharmacist prescribing: a scoping review comparing the UK, New Zealand, Canadian and Australian experiences. Int J Pharm Pract. 2019. 10.1111/ijpp.12557. 31287208 10.1111/ijpp.12557

[60] Medication reconciliation as a strategic priority. Accreditation Canada. 2022. https://physicians.nshealth.ca/sites/default/files/2022-05/NSHealth%20Medication%20Reconciliation%20One%20Pager.pdf. Accessed 26 Mar 2025.

[61] The patient-centred medical home: integrating comprehensive medication management to optimize patient outcomes: resource guide. In: Patient-Centred Primary Care Collaborative. 2nd ed. 2012. www.pcpcc.org/sites/default/files/media/medmanagement.pdf. Accessed 26 Mar 2025.

[62] Yeheskel A, Rawal S. Exploring the ‘patient experience’ of individuals with limited English proficiency: a scoping review. J Immigr Minor Health. 2019. 10.1007/s10903-018-0816-4. 30203377 10.1007/s10903-018-0816-4

[63] Al Shamsi H, Almutairi AG, Mashrafi SA, et al. Implications of language barriers for healthcare: a systematic review. Oman Med J. 2020. 10.5001/omj.2020.40. 32411417 10.5001/omj.2020.40 PMC7201401

[64] Naef R, Ernst J, Bürgi C, et al. Quality of acute care for persons with cognitive impairment and their families: a scoping review. Int J Nurs Stud. 2018. 10.1016/j.ijnurstu.2018.05.006. 29859348 10.1016/j.ijnurstu.2018.05.006

[65] Velmans SR, Joseph C, Wood L, et al. A systematic review and thematic synthesis of inpatient nursing staff experiences of working with high-risk patient behaviours. J Psychiatr Ment Health Nurs. 2023. 10.1111/jpm.12987. 10.1111/jpm.12987 37874310

