# Supplementary material for: Perspectives on potential pharmacist prescribing in an outpatient dialysis center: qualitative interviews with patients and clinicians
Source: Int J Clin Pharm. 2026 Jan 17;48(3):886–96. doi: 10.1007/s11096-025-02084-x (PMC13176218; doi:10.1007/s11096-025-02084-x)
Supplement: Supplementary file 1 — Supplementary file1 (PDF 226 KB) [file 11096_2025_2084_MOESM1_ESM.pdf]

Article title: Perspectives on pharmacist prescribing in an outpatient dialysis center: Qualitative interviews with patients and clinicians

Journal: International Journal of Clinical Pharmacy

Author names: Angela S. Choi, Madeline Theodorlis, Angelina Abbaticchio, Marisa Battistella

Corresponding author: Marisa Battistella[a,b]

Affiliations:

a. Leslie Dan Faculty of Pharmacy, University of Toronto, Toronto, Ontario, Canada
b. Toronto General Hospital, University Health Network, Toronto, Ontario, Canada

Email: marisa.battistella@uhn.ca

Online Resource 1. Patient Interview Guide

INTRODUCTION

My name is *state name* and I am a Research Coordinator/Student working with [pharmacist name], a pharmacist in the hemodialysis unit. Before we start, I would like to remind you that this interview will be audio recorded and transcribed. I will let you know when I start recording. Please avoid using personal identifying information, like names, where possible. When we transcribe the interviews, we will remove any identifying information to ensure confidentiality. During this interview, you may become uncomfortable while discussing your experiences. You may refuse to answer questions or leave/stop the interview at any time if you experience any discomfort. This whole process should take about 15-20 minutes. If you have any questions throughout the interview, please let me know.

STUDY BACKGROUND

Prescribing medications is usually done by medical practitioners, like physicians. However, pharmacists are well-equipped to prescribe medications because of their training in managing diseases, checking for drug interactions, and monitoring side effects. As you may know, in some places, like [province name], pharmacists are able to prescribe medications for minor ailments like urinary tract infections, which means you can receive and fill a prescription right at your local pharmacy. However, in [province name], pharmacists are not authorized to prescribe medications **within the hemodialysis unit**.

We're interested in hearing your thoughts on whether pharmacists should be allowed to prescribe in hemodialysis units, and how you think that might affect your care. When we say "prescribing", this could mean pharmacists writing a prescription for a minor ailment or adjusting the dose of a medication, such as blood pressure medications. For example, pharmacists in the hemodialysis unit prescribing pink eye or heartburn, if patients were to come in for their dialysis session with such conditions.

Before we begin recording, we want to ask a couple of questions about yourself. This information will only be associated with your study identification number, and not your name:

- What is your current marital status?
- What is the highest level of education you've received?
- How would you describe your ethnicity?

To ensure your voice is picked up by the audio recording, please speak clearly and loudly.
Do you have any questions before we begin the recording?

I will start recording now.

Start recording

INTERVIEW QUESTIONS

My first few questions are about your experience with receiving and filling prescriptions.

1) **Who currently prescribes your medications?**
   Examples: family doctor, nurse practitioner, nephrologist, cardiologist, or another specialist
   Prompt: Is it easy to get prescriptions?
   - IF YES: What makes it easy?
   - IF NO: What would have made the experience [specific experience, if mentioned] better?

2) **Who usually makes changes to your medications?**
   Prompt: Is it easy to get prescriptions?
   - IF YES: What makes it easy?
   - IF NO: What would have made the experience [specific experience, if mentioned] better?

3) **What challenges do you face when trying to get a prescription or medication refill?**
   Prompt:
   - IF NO CHALLENGES: What makes it easy for you to get a prescription or refill?
   - IF CHALLENGES: How does this affect you and/or your ability to get the medications you need?
     - What would make it easier for you to get a prescription or refill?
       Example: Having other healthcare providers, such as pharmacists or nurses, be able to prescribe.

My next questions are about your experience with pharmacists in the hemodialysis unit.

4) **In your view, what do you think pharmacists' role is in the hemodialysis unit?**
   Prompts:
   - How do you think pharmacists contribute to your care here/in the dialysis unit?
   - Have they ever helped you understand your medication?

   Example (IF UNSURE): their main role is to optimize medication use by checking for interactions between drugs, monitoring for side effects, and offering medication counseling.

5) **How often do you interact with pharmacists in the hemodialysis unit?**
   Prompts:
   - Can you describe your interactions with pharmacists in the dialysis unit? Was the interaction helpful?
   - Do you think it would be helpful to have more interactions with pharmacists in the dialysis unit? Why or why not?

6) **Have you ever had a pharmacist write you a prescription at your local pharmacy before?**
   Examples: Currently, [province name] pharmacists can prescribe medications for 19 minor ailments such as pink eye, urinary tract infection, etc.

Prompts:
- IF YES: What was your experience like?
- IF NO: Is this something you would be comfortable with? Why or why not?

The last few questions are about your views on pharmacists prescribing in the hemodialysis unit.

7) **How would you feel about pharmacists being able to prescribe medications in the hemodialysis unit?**

   Prompts:
   - How would you feel about pharmacists making changes to your medications in the hemodialysis unit such as adjusting the dose or frequency of a medication?
   - How would you feel about pharmacists prescribing medications for minor ailments in the hemodialysis unit?
   - What might make you feel more comfortable?
     - Examples: prescriptions being approved or reviewed by another healthcare provider (e.g., nephrologist).
   - If "trust" is mentioned: What makes you trust the [specific clinician] that prescribes medications for you?

8) **How do you think pharmacists prescribing in the hemodialysis unit would make a difference in your care?**

   Examples:
   - Help address medication-related issues faster.
   - Provide better/more comprehensive explanations about prescriptions.
   - Reduce potentially conflicting advice from different healthcare providers.

9) **If pharmacists were to start prescribing medications in the hemodialysis unit, how would you want to know about this information?**

   Prompts:
   - What kind of information would you want to know about this change?
   - How should other healthcare providers be involved in communicating this change?

   Examples:
   - Have a trusted healthcare provider (doctor, nurse practitioner, etc.) explain this change to you.
   - A pamphlet or video explaining the change.

That is the end of the interview questions. Is there anything else you'd like to share before we stop recording?

CONCLUSION

Thank you for taking the time to share your experiences. Your input is very valuable to us.

Stop recording
