# Supplementary material for: Perspectives on potential pharmacist prescribing in an outpatient dialysis center: qualitative interviews with patients and clinicians
Source: Int J Clin Pharm. 2026 Jan 17;48(3):886–96. doi: 10.1007/s11096-025-02084-x (PMC13176218; doi:10.1007/s11096-025-02084-x)
Supplement: Supplementary file 2 — Supplementary file2 (PDF 200 KB) [file 11096_2025_2084_MOESM2_ESM.pdf]

Article title: Perspectives on pharmacist prescribing in an outpatient dialysis center: Qualitative interviews with patients and clinicians

Journal: International Journal of Clinical Pharmacy

Author names: Angela S. Choi, Madeline Theodorlis, Angelina Abbaticchio, Marisa Battistella

Corresponding author: Marisa Battistella[a,b]

Affiliations:

a. Leslie Dan Faculty of Pharmacy, University of Toronto, Toronto, Ontario, Canada
b. Toronto General Hospital, University Health Network, Toronto, Ontario, Canada

Email: marisa.battistella@uhn.ca

Online Resource 2. Clinician Interview Guide

INTRODUCTION

My name is *state name* and I am a Research Coordinator/Student working with [pharmacist name]. Before we start, I would like to remind you that this interview will be audio recorded and transcribed. I will let you know when I start recording. Please avoid using personal identifying information where possible. When we transcribe the interviews, we will remove any identifying information to ensure confidentiality. During this interview, you may become uncomfortable while discussing your experiences. You may refuse to answer questions or leave/stop the interview at any time if you experience any discomfort. This whole process should take about 15-20 minutes. If you have any questions before we start, please let me know.

STUDY BACKGROUND

Prescribing medications is usually done by medical practitioners. However, pharmacists are well-equipped to act as non-medical prescribers because of their knowledge of, and training in pharmacology, pharmacokinetics, disease management, drug interactions, and adverse events. As you may know, in [province name], pharmacists are now authorized to prescribe medications for minor ailments like urinary tract infections and are able to provide and fill a prescription within local pharmacies. However, pharmacists in [province name] do not have permissions to prescribe **within the hemodialysis unit.**

The purpose of this interview is to understand your thoughts on whether pharmacists should be able to prescribe in the hemodialysis unit, and how it may impact patient care and clinical workflow. When we say “prescribing”, this could mean pharmacists writing a prescription for a minor ailment or adjusting the dose of a medication, such as blood pressure medications.

Before we begin recording, we would like to collect some demographic information about you. As a reminder, this information will be confidential and will only be associated with your study number.

- What is your age?
- What is your gender?
- How would you describe your ethnicity?
- What is your clinical role?
- How many years of clinical experience do you have?

To ensure your voice is picked up by the audio recording, please speak clearly and loudly. Do you have any questions before we begin the recording?

I will start the recording now.

Start recording

INTERVIEW QUESTIONS

1) **Who currently prescribes medications for patients in the hemodialysis unit, and what does that process typically involve?**

    <u>Prompts</u>:
    - What are the strengths and weaknesses of the current prescribing workflow?

2) **In your view, what is the role of pharmacists in the hemodialysis unit?**

    <u>Prompts</u>:
    - How do pharmacists currently contribute or fit into the current prescribing workflow here/in the hemodialysis unit?
    - How do pharmacists contribute to patient care and medication management?

3) **What are your thoughts on pharmacists prescribing medications in the hemodialysis unit?**

    <u>Prompts</u>:
    - Do you foresee any barriers or challenges with this change?
    - How could these be addressed?

    <u>Examples</u>:
    - Concerns about potential overlaps or conflicts in roles and responsibilities with other prescribers in the unit.
    - Patient and/or clinician resistance/hesitancy.

4) **In what ways might the <u>hemodialysis team</u> benefit from pharmacists prescribing in the hemodialysis unit?**

    <u>Prompts</u>:
    - How would this impact workflow efficiency or workload distribution?
    - How might this impact interprofessional collaboration or affect the roles of other clinicians in the unit?

5) **In what ways might <u>patients</u> benefit from pharmacists prescribing in the hemodialysis unit?**

    <u>Examples</u>:
    - Provide opportunity for more education around medications.
    - More convenient or faster access to medications.
    - Address medication-related issues (e.g., non-adherence, inappropriate doses).

    <u>Prompts</u>:
    - How do you think pharmacists prescribing in the hemodialysis unit would make a difference in patient education and understanding of their medications?

6) **If pharmacists were to be permitted to prescribe in the hemodialysis unit, how do you think this should be implemented?**

    <u>Prompts</u>:
    - What skills or training do you feel are critical for pharmacists to have before taking on prescribing responsibilities?
    - What would help you feel confident in their role as prescribers?

7) **If pharmacists were to be permitted to prescribe in the hemodialysis unit, how do you think this should be communicated to patients?**

Prompts:

- How should other healthcare providers be involved in communicating this change?

Examples:

- Have a trusted healthcare provider (doctor, nurse practitioner, etc.) explain this change to you.
- A pamphlet or video explaining the change.

That is the end of the interview questions. Is there anything else you'd like to share before I stop the recording?

CONCLUSION

Thank you for taking the time to share your experiences. Your input is very valuable to us.

Stop recording
